# Supplementary figures and images for: Mitochondrial Aurora kinase A induces mitophagy by interacting with MAP1LC3 and Prohibitin 2
Source: Life Sci Alliance. 2021 Apr 5;4(6):e202000806. doi: 10.26508/lsa.202000806 (PMC8046421; doi:10.26508/lsa.202000806)

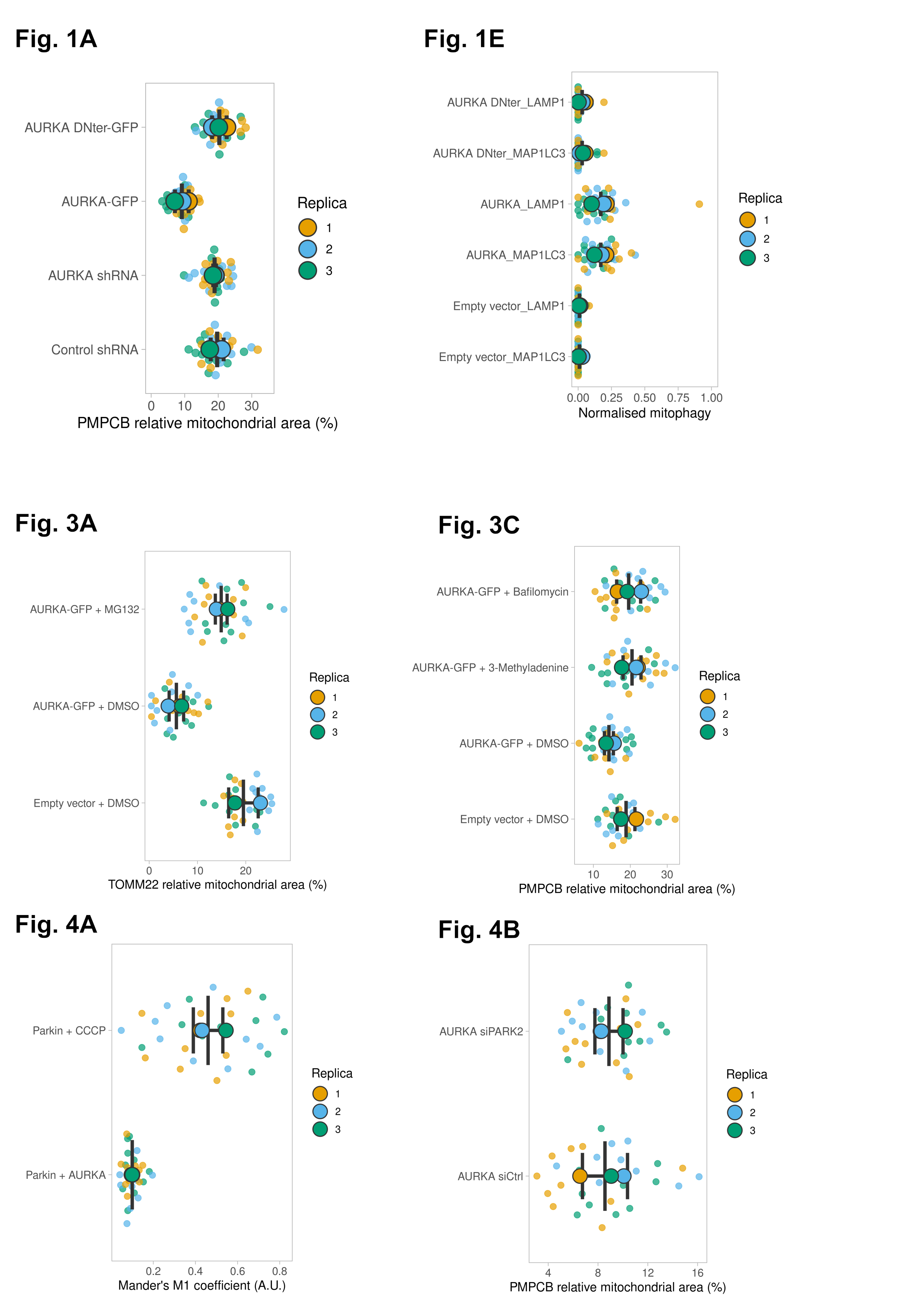

Supplement: Supplementary file 1 [file LSA-2020-00806_SdataF1_F3_F4.tif]

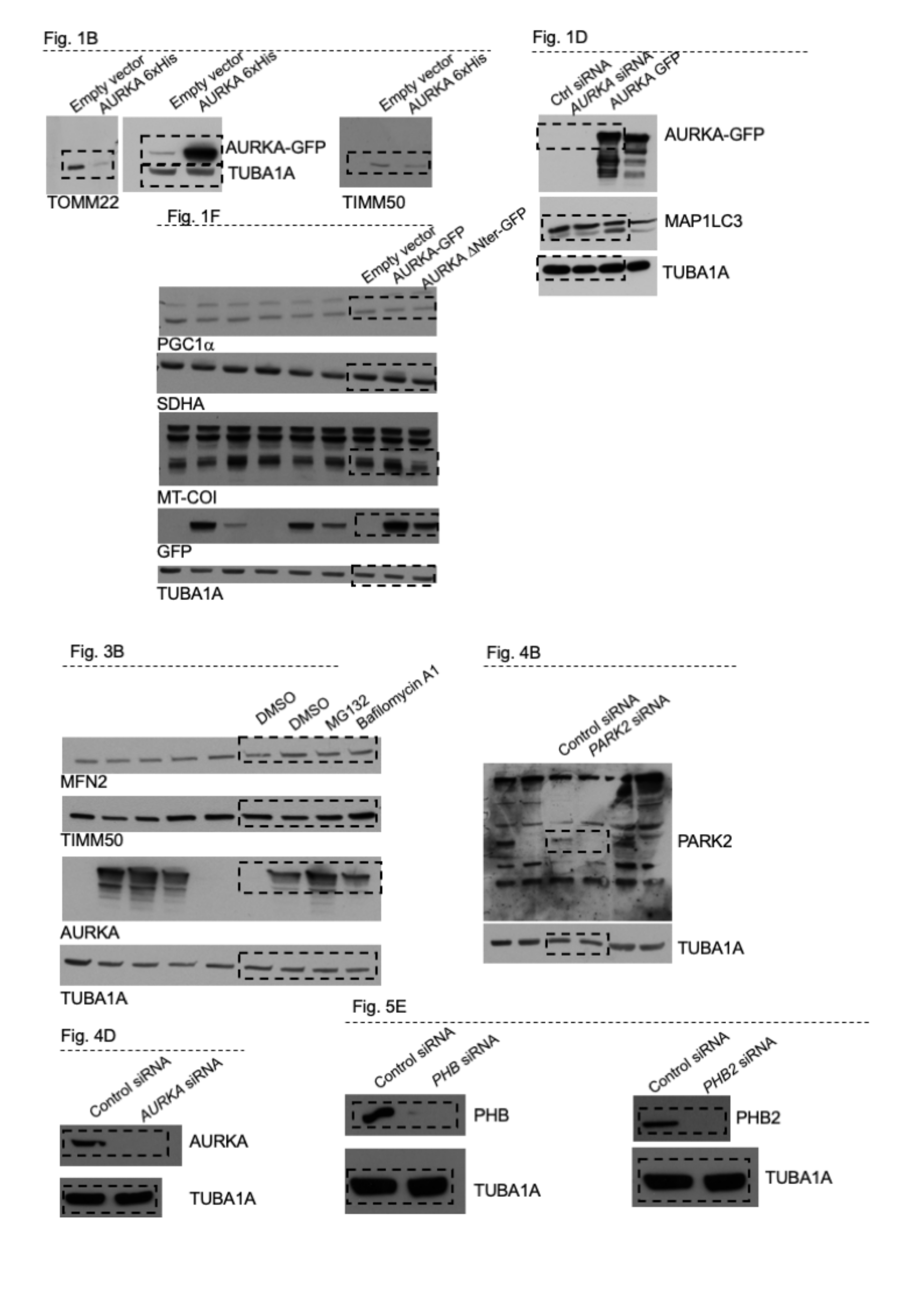

Supplement: Supplementary file 2 [file LSA-2020-00806_SdataF1_F3_F4_F5.tif]

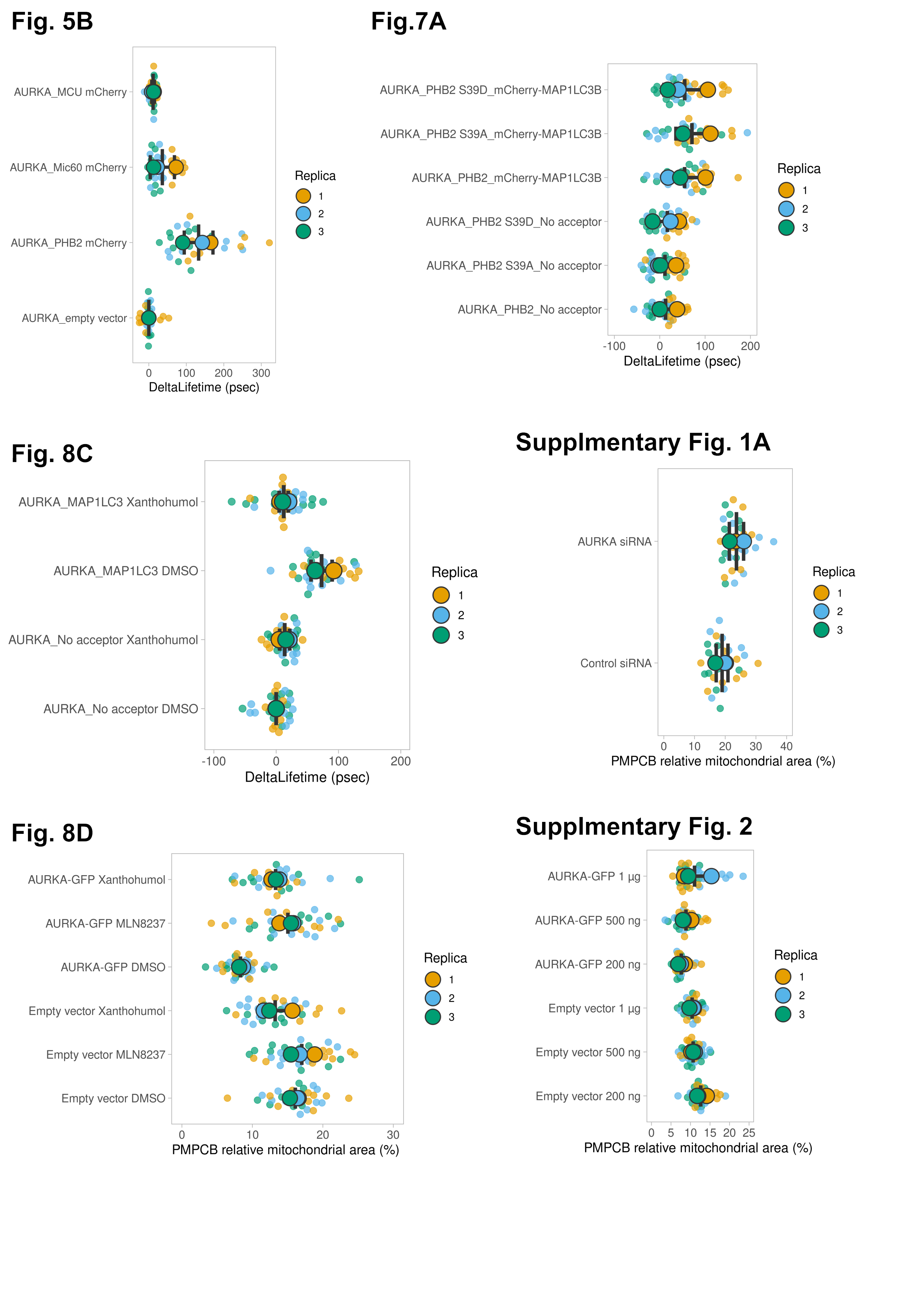

Supplement: Supplementary file 3 [file LSA-2020-00806_SdataF5_F7_F8_FS1_FS2.tif]

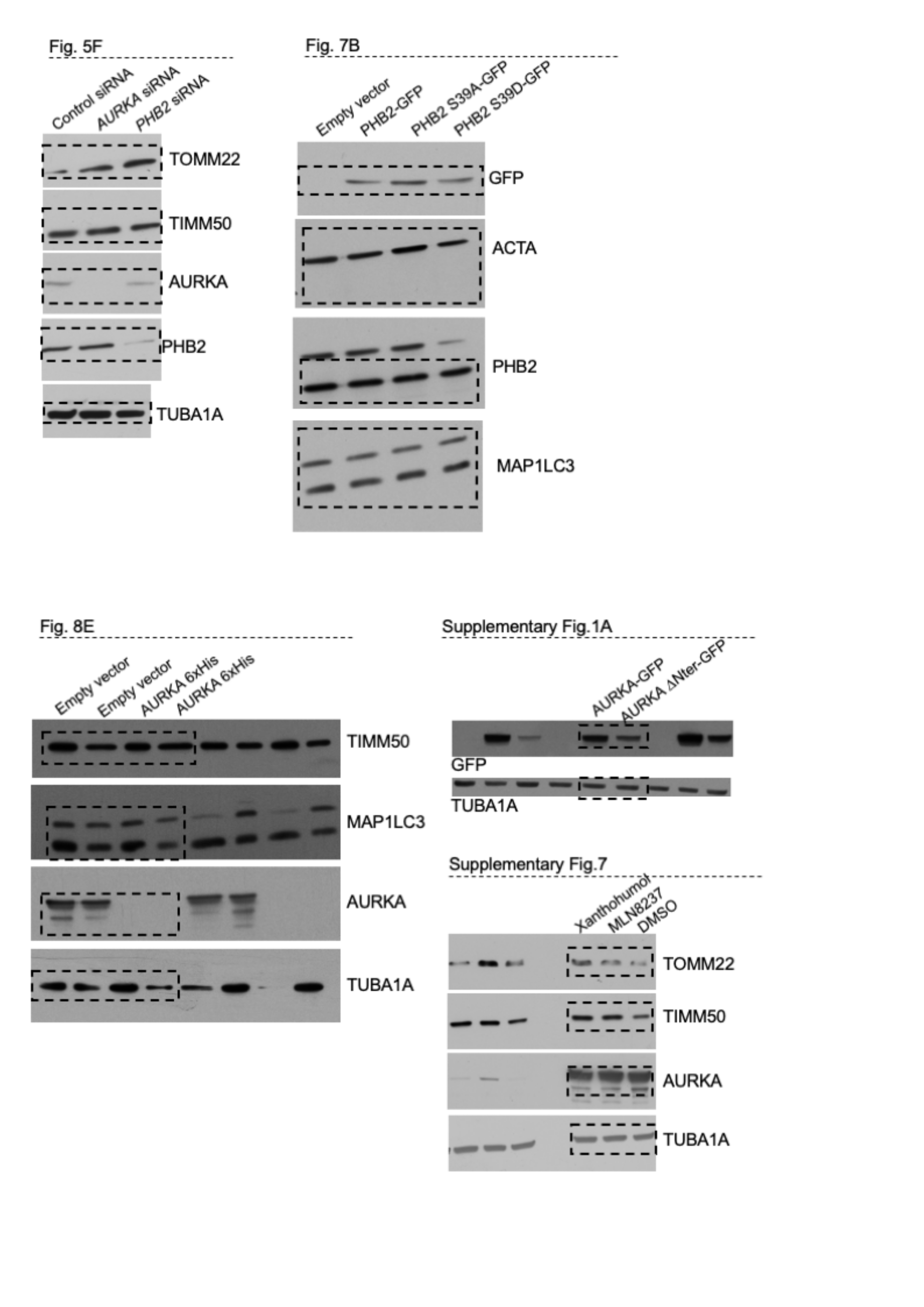

Supplement: Supplementary file 4 [file LSA-2020-00806_SdataF5_F7_F8_FS1_FS7.tif]
