# Supplementary material for: Mitochondrial Aurora kinase A induces mitophagy by interacting with MAP1LC3 and Prohibitin 2
Source: Life Sci Alliance. 2021 Apr 5;4(6):e202000806. doi: 10.26508/lsa.202000806 (PMC8046421; doi:10.26508/lsa.202000806)
Supplement: Supplementary file 7 [file LSA-2020-00806_Supplemental_Data_1.docx]

# SourceData Captions

**Source data Fig. 1.** Representative uncropped blots. Scans of the full blots used in the study, along with each detected protein and the corresponding figure. The areas enclosed by a dashed rectangle are the portions of the blots integrated in the figure of the study.

**Source data Fig. 2.** Representative uncropped blots. Scans of the full blots used in the study, along with each detected protein and the corresponding figure. The areas enclosed by a dashed rectangle are the portions of the blots integrated in the figure of the study.
